# Exploratory analysis of associations among weight loss, oral function, and dysgeusia in patients with lung cancer undergoing chemotherapy

**DOI:** 10.1186/s12903-025-07460-7

**Published:** 2025-12-11

**Authors:** Utae Katsushima, Satoshi Kurose, Takuya Fukushima, Kazuki Fujii, Yutaro Nagata, Yume Sanada, Kiyori Yoshida, Tatsuki Ikoma, Yuki Takeyasu, Yuta Yamanaka, Takayasu Kurata

**Affiliations:** 1 Department of Thoracic Oncology, Kansai Medical University Hospital, 2-3-1 Shin-machi, Hirakata-city, Osaka, 573-1191 Japan; 2 Faculty of Sport and Health Sciences, Osaka Sangyo University, 3-1-1, Nakagaito, Daito-city, Osaka, 573-8530 Japan; 3 Faculty of Rehabilitation, ansai Medical University Hospital, 18-89 Uyamahigashi-cho, Hirakata-city, Osaka, 573-1136 Japan

**Keywords:** Oral frailty, Dysgeusia, Weight loss, Lung cancer, Chemotherapy, Nutritional status

## Abstract

**Background:**

Cancer-related weight loss is associated with poor prognosis, reduced treatment tolerance, and impaired quality of life. Recent studies have highlighted the importance of oral health in maintaining nutritional status among older adults. Oral frailty, a reversible stage of oral function decline, has been linked to reduced food intake and systemic frailty. However, the relationship between oral frailty and weight loss in patients with lung cancer undergoing chemotherapy remains unclear. This study aimed to identify factors associated with weight loss, with a particular focus on oral frailty, in this population.

**Methods:**

This single-center retrospective study included 67 patients with lung cancer who received outpatient chemotherapy at Kansai Medical University Hospital between October and December 2024. Oral frailty was evaluated using the 5-item Oral Frailty Checklist (OF-5) by non-dental clinicians on the day of chemotherapy. Weight loss was defined as an unintentional loss of > 5% within 6 months. Logistic regression models were constructed to examine associations between oral frailty, dysgeusia, and weight loss, adjusting stepwise for performance status, C-reactive protein, sex, and cancer stage. Model robustness was confirmed using bootstrap resampling (1,000 replications).

**Results:**

Nineteen patients (28.4%) experienced significant weight loss. In multivariate analyses, oral frailty (odds ratio [OR]: 28.79, 95% confidence interval [CI]: 1.81–36.05, *p* = 0.006) and dysgeusia (OR: 39.84, 95% CI: 1.76–40.62, *p* = 0.001) were independently associated with weight loss after adjustment for confounders. These associations remained robust in bootstrap analyses. No multicollinearity was detected among variables.

**Conclusions:**

Oral frailty and dysgeusia were significantly associated with weight loss in patients with lung cancer receiving chemotherapy. Screening for oral frailty using the simple OF-5 checklist, even when performed by non-dental clinicians, may facilitate early identification of patients at risk for nutritional decline. Multidisciplinary supportive care, including oral and taste function management, should be integrated into oncology practice to help mitigate cancer-related weight loss.

## Background

Cancer remains a major public health challenge and has been the leading cause of death in Japan since 1981 [1]. Weight loss is frequently observed among patients with cancer, and anorexia, malnutrition, and weight loss are each strongly associated with poor prognosis, reduced treatment tolerance, and deterioration in quality of life (QOL) [2, 3]. Multiple factors contribute to weight loss, including pain, gastrointestinal symptoms, brain metastases, chemotherapy-induced nausea, and psychological factors; therefore, early identification and intervention are critically important in clinical practice [4].

Previous research suggests that, among older adults, oral health is significantly associated with weight loss [5]. In community-dwelling older men, the number of remaining teeth and appetite have been reported to be independently associated with weight change [6]. The impact of oral health and function on nutritional status has therefore received increasing attention [5, 6].

Oral frailty refers to a reversible, intermediate stage between a healthy oral condition and overt oral functional decline, characterized by mild impairments in mastication, swallowing, speech, and oral hygiene [7, 8]. Symptoms may include food spillage, dysarthria, and mild eating difficulty, and oral frailty has been reported in approximately 40% of older adults. Pathophysiologically, oral frailty and systemic frailty may interact bidirectionally. Systemic frailty, characterized by reduced physical activity, undernutrition, and psychosocial decline, may precipitate oral functional decline through masticatory muscle sarcopenia, hyposalivation, and impaired oromotor coordination. Conversely, oral frailty may promote inadequate dietary intake and malnutrition, thereby accelerating weight loss and physical decline and worsening systemic frailty [9–13]. Thus, oral frailty represents both a consequence and a driver of systemic frailty, and because it is mild and potentially reversible, early detection and intervention are essential [12–15].

Most previous research on oral frailty has focused on community-dwelling older adults. Among patients with cancer, studies have primarily targeted head and neck or oral cancers, often focusing on mucositis and dysphagia management [16–18]. In contrast, Li et al. conducted a cross-sectional study across multiple cancer types, including lung, gastric, and gynecologic cancers, and assessed oral frailty using the Oral Frailty Index-8 (OFI-8). They reported a high prevalence (57.6%) and implicated multifactorial contributors such as xerostomia, denture use, dysgeusia, and psychosocial factors [19]. Teranishi et al. further reported that reduced functional tooth number and oral frailty were important risk factors for postoperative weight loss in patients with gastric cancer undergoing gastrectomy [9].

Lung cancer has particularly high incidence and mortality and is associated with poor prognosis [20, 21]. It is also one of the most common malignancies in older populations [21, 22]. Lung cancer is often diagnosed at an advanced stage, for which chemotherapy is a mainstay treatment [23, 24]. Weight loss in patients with lung cancer receiving chemotherapy appears to be influenced by diverse factors, and it remains unclear which factors, including oral frailty, are most strongly associated with weight loss. Identifying such factors may facilitate earlier intervention, which is important given the prognostic significance of cancer-related weight loss.

The Oral Frailty Index-5 (OF-5) is a newly proposed five-item checklist (2024 joint statement by the Japan Geriatrics Society, the Japanese Society of Gerodontology, and the Japan Society of Sarcopenia and Frailty) that enables rapid screening for oral frailty by non-dental clinicians [7, 25]. Although cross-cancer studies exist, reports focusing on patients with lung cancer undergoing chemotherapy and using the OF-5 remain limited. Therefore, we hypothesized that oral frailty may be associated with weight loss during chemotherapy for lung cancer. The present study examined factors associated with weight loss, focusing particularly on oral frailty assessed using the OF-5, in patients with lung cancer undergoing chemotherapy.

## Methods

### Study design and participants

We conducted a single-center, retrospective observational study of patients with lung cancer who received outpatient chemotherapy in the Department of Respiratory Oncology at Kansai Medical University Hospital between October and December 2024. Consecutive patients who underwent oral frailty screening using the OF-5 during the observation period were identified. Patients with thoracic malignancies other than lung cancer were excluded. The sample size was determined by all eligible cases within the study period (convenience sample); no a priori sample-size calculation was performed.

### Index date and timing of measurements

The index date was defined as the day on which the OF-5 was administered by the treating outpatient physician during a chemotherapy visit. Body weight, handgrip strength, performance status (PS) according to the Eastern Cooperative Oncology Group (ECOG), and laboratory parameters (including C-reactive protein [CRP] and albumin [Alb]) were obtained on the same day. Symptoms (dysgeusia, xerostomia, oral mucositis, nausea) and prescription histories (topical oral agents for mucositis, analgesics, proton pump inhibitors [PPIs], steroids, non-steroidal anti-inflammatory drugs [NSAIDs], and oral nutritional supplements) were determined by chart review covering the 7 days preceding the index date. Dental visits within the prior 6 months were also recorded.

### Outcome

The primary outcome was unintentional weight loss over the preceding 6 months. Weight loss was calculated using the weight measured on the index date and the self-reported or chart-documented maximum weight within the prior 6 months, as follows:

$$\:\frac{maximum\:weight\:within\:6\:months-index\:weight}{maximum\:weight\:within\:6\:months}\times\:100\left(\%\right)$$

Weight loss ≥ 5% was considered significant [26].

### Exposures and covariates

#### Oral frailty (OF-5; Table 1):

The OF-5 includes five items: reduced number of remaining teeth, difficulty in mastication, difficulty in swallowing, oral dryness, and impaired lingual or articulatory function. The presence of ≥ 2 positive items indicates oral frailty [7, 26] (Table 1). The OF-5 was administered by non-dental clinicians during a chemotherapy visit; no simultaneous dental examination was performed. OF-5, weight, handgrip strength, PS, CRP, and Alb were obtained on the index date (or a closely adjacent date), whereas dysgeusia and oral mucositis were assessed based on information from the preceding 7 days.

**Table 1:** Oral frailty 5-item checklist

Component	Questionnaire Item	Response (Applicable)	Response (Not applicable)
Fewer teeth	How many of your natural teeth are left?	0–19 teeth	≥ 20 teeth
Difficulty in chewing	Do you have any difficulties in eating tough foods now compared to 6 months ago?	Yes	No
Difficulty in swallowing	Have you choked on your tea or soup recently?	Yes	No
Dry mouth	Do you often experience dryness of mouth?	Yes	No
Low articulatory oral motor skill*	Have you had any difficulty in clear pronunciation recently?	Yes	No

Component	Measurement	Response (Applicable)	Response (Not applicable)
Low articulatory oral motor skill*	Oral DDK with/ta/	< 6.0 times/s	≥ 6.0 times/s

If the answer is “yes” for each item, one point is given, and the total score (ranging from 0 to 5) is calculated. A score of 2 or more indicates oral frailty

*Low articulatory oral motor skill can be assessed by the oral diadochokinesia (oral DDK) test

#### Dysgeusia

Dysgeusia was assessed in the outpatient chemotherapy clinic according to the Common Terminology Criteria for Adverse Events (CTCAE) v5.0. Grades 0–1 were categorized as no dysgeusia, and grades 2–3 were categorized as dysgeusia.

#### Appetite

Appetite was assessed using a single item aligned with item A (reduced food intake) of the Mini Nutritional Assessment–Short Form (MNA-SF) [27]. The total MNA-SF score and categorical classifications were not used. Scores of 0 (marked decrease) or 1 (moderate decrease) were classified as appetite loss; a score of 3 (no decrease) was classified as no appetite loss.

#### Oral symptoms and medications

The presence of oral mucositis and nausea during the preceding 7 days was confirmed by the treating physician using patient questionnaires and clinical interviews. Prescriptions for topical oral agents or oral care products for mucositis, mucosal protective agents, PPIs, steroids, NSAIDs, and nutritional supplements were obtained from medical records.

#### Physical function, nutrition, and inflammation

Handgrip strength was measured bilaterally using a hand dynamometer (GRIP-D; Takei Scientific Instruments Co., Ltd., Niigata, Japan) on the index day, and the maximal value was used. Low grip strength was defined as < 28 kg for men and < 18 kg for women [28]. ECOG PS was recorded, and CRP and Albumin values were obtained on the index date or the closest available date. Body mass index (BMI) was calculated as weight/height² using measurements from the index visit.

#### Tumor-related variables

Histology, stage, and treatment regimen were obtained from the medical record. Disease status at the time of oral frailty assessment was determined using the most recent imaging evaluation according to Response Evaluation Criteria in Solid Tumors (RECIST), version 1.1. Although no dental examination was performed on the index date, data from prior visits to the hospital’s oral and maxillofacial surgery department (e.g., for preoperative assessment or bisphosphonate therapy evaluation) were included as reference information.

### Handling of missing data

Analyses were performed using complete-case analysis. The analytic cohort comprised patients with available data on the outcome and the main exposures (OF-5 and dysgeusia).

### Statistical analysis

Continuous variables were presented as mean ± standard deviation or median (interquartile range), and categorical variables, as counts (percentages). Normality was tested using the Shapiro–Wilk test. Between-group comparisons were conducted using the t-test or Mann–Whitney U test, as appropriate, and proportions were compared using the χ² test.

The primary analysis used logistic regression with weight loss (yes/no) as the dependent variable. To avoid overfitting and estimation bias due to excessive covariate inclusion, model specification focused on clinically important and relatively independent variables, with oral frailty and dysgeusia as the primary exposures of interest. Variables associated with weight loss in univariable analyses were explored, and robustness was examined through stepwise adjustment and bootstrap resampling. The logistic regression models were specified as follows:

Model 1: Oral frailty and dysgeusia.

Model 2: Model 1 + performance status and CRP, selected as principal confounders reflecting disease burden and inflammation.

Model 3: Model 2 + sex and cancer stage, included as standard demographic and disease characteristics.

Appetite loss was excluded from the primary models due to conceptual overlap with dysgeusia. Handgrip strength and BMI were evaluated in sensitivity or supplementary analyses because of events-per-variable (EPV) constraints and their potential role as mediators or redundant covariates.

Variance inflation factors (VIFs) were examined, with VIF > 5 considered suggestive of multicollinearity. Because BMI reflects baseline nutritional status but may lie on the causal pathway from reduced intake to weight loss, BMI was excluded from the primary models to avoid overadjustment and was examined only in the sensitivity analyses. Model uncertainty was assessed using bootstrap resampling with 1,000 iterations. A fixed random seed (12345) was used to ensure reproducibility. A simple (non-stratified) resampling approach was used. Confidence intervals were estimated using the percentile method, and two-sided p-values with 95% confidence intervals (CIs) were reported. Two-sided tests were used with a significance level of 0.05. All analyses were performed using SPSS Statistics 26.0 J (IBM).

### Ethics approval and consent to participate

This study was approved by the Institutional Review Board of Kansai Medical University (Approval No. 2023264) and conducted in accordance with the Declaration of Helsinki.

Given the single-center, retrospective nature of this study, which utilized de-identified clinical data collected during routine care, the Institutional Review Board waived the requirement for individual written informed consent. Instead, an opt-out consent procedure was implemented on the department’s webpage of the university hospital. Study information was publicly disclosed, providing all eligible patients with comprehensive details about the study and the opportunity to decline participation. Patients who did not express refusal were considered to have consented. No patients opted out. All personal information was anonymized prior to analysis.

## Results

### Frequency of weight loss and baseline characteristics (Table 2)

Among 72 patients with lung cancer who received outpatient chemotherapy during the observation period, 67 were included in the analysis after excluding those with incomplete records or other thoracic malignancies. The median age was 73.0 years (interquartile range [IQR]: 66.5–76.0); 50 patients (74.6%) were men and 17 (25.4%) were women.

**Table 2:** Patient characteristics (*N* = 67)

Variable	All (N = 67)	BWL (*n* = 19)	Non-BWL (*n* = 48)	*p*-value
Age, median (IQR), years	73 (66.5–76.0)	71.0 (68.0–76.0)	73.0 (60.5–76.7)	0.967
Sex, n (%)				0.609
Male	50 (74.6)	15 (78.9)	35 (72.9)	
Female	17 (25.4)	4 (21.1)	13 (27.1)	
Performance Status (PS), n (%)				< 0.001
0	19 (28.4)	0 (0.0)	19 (39.6)	
1	34 (50.7)	10 (52.6)	24 (50.0)	
2	14 (20.9)	9 (47.4)	5 (10.4)	
Pathology, n (%)				0.432
Adenocarcinoma	29 (43.3)	7 (36.8)	21 (43.8)	
Squamous cell carcinoma	13 (19.4)	3 (15.8)	10 (20.8)	
Small-cell carcinoma	21 (31.3)	8 (42.1)	13 (27.1)	
Other	4 (6.0)	1 (5.3)	4 (8.3)	
Stage, n (%)				0.548
I	3 (4.5)	1 (5.3)	2 (4.2)	
II	6 (9.0)	4 (21.1)	2 (4.2)	
III	16 (23.8)	3 (15.8)	13 (27.1)	
IV	33 (49.3)	9 (47.4)	24 (50.0)	
Recurrence	9 (13.4)	2 (10.4)	7 (14.5)	
Genetic mutation, n (%)				0.839
None	21 (31.3)	5 (26.3)	16 (33.3)	
EGFR	7 (10.4)	1 (5.3)	6 (12.5)	
KRAS	6 (9.0)	2 (10.5)	4 (8.3)	
BRAF	2 (3.0)	1 (5.3)	1 (2.1)	
MET skipping	1 (1.5)	0 (0.0)	1 (3.2)	
Other	1 (1.5)	0 (0.0)	1 (2.1)	
Unknown	29 (43.3)	10 (52.6)	19 (39.6)	
PD-L1 Tumor Proportion Score, n (%)				0.516
<1%	9 (13.4)	2 (10.5)	7 (14.6)	
1–49%	11 (16.4)	4 (21.1)	7 (14.6)	
≥50%	14 (20.9)	2 (10.5)	12 (25.0)	
Unknown	33 (49.3)	11 (57.9)	22 (45.8)	
Treatment line, n (%)				0.874
First line	23 (34.4)	8 (42.1)	15 (31.3)	
Second line	11 (16.4)	3 (15.8)	8 (16.7)	
Third line or more	12 (17.9)	3 (15.8)	9 (18.6)	
Adjuvant chemotherapy	14 (20.9)	4 (21.1)	10 (20.8)	
Chemoradiotherapy	7 (10.4)	1 (5.3)	6 (12.5)	
Treatment, n (%)				ICI, yes:0846 CA, yes:0.150
CA** + ICI	12 (17.9)	6 (31.6)	6 (12.5)	
*** Chemotherapy alone (CA)	25 (37.3)	7 (36.8)	18 (37.5)	
Immune checkpoint inhibitor (ICI)	22 (32.8)	4 (21.1)	18 (37.5)	
Molecular targeted therapy	1 (1.5)	0 (0.0)	1 (2.1)	
****CA + molecular targeted therapy	7 (10.5)	2 (10.5)	5 (10.4)	
Disease status, n (%)				0.574
CR	0 (0.0)	0 (0.0)	0 (0.0)	
PR	40 (59.7)	11 (57.9)	29 (60.4)	
SD	14 (20.9)	3 (15.8)	11 (22.9)	
PD	13 (19.4)	5 (26.3)	8 (16.7)	
BMI, median (IQR), kg/m²	23.02 ± 3.91	24.82 ± 4.37	22.38 ± 3.48	0.020
Handgrip weakness (Male < 28 kg, Female < 18 kg), n (%)	27 (40.3)	14 (73.7)	13 (27.1)	< 0.001
Appetite loss, yes, n (%)	21 (31.3)	11 (57.9)	10 (20.8)	0.003
CRP, median (IQR), mg/dL	0.68 (0.13–1.23)	1.12 (0.38–3.41)	0.23 (0.09–0.79)	0.004
Alb, median (IQR), mg/dL	3.9 [2.9–5.8]	3.9 [3.4–4.2]	3.9 [3.8–4.2]	0.527
Oral frailty, yes, n (%)	31 (46.3)	16 (84.2)	15 (31.3)	< 0.001
*Dental visit, yes, n (%)	42 (62.7)	13 (68.4)	29 (60.4)	0.541
Dentures, yes, n (%)	31 (46.3)	9 (47.4)	22 (45.8)	0.910
Periodontitis, n (%)				0.416
Yes	45 (67.2)	14 (73.7)	31 (64.6)	
No	4 (6.0)	0 (0.0)	4 (8.3)	
Unknown	18 (26.9)	5 (26.3)	13 (27.1)	
Ill-fitting dentures, n (%)				0.365
Yes	9 (13.4)	1 (5.3)	8 (16.7)	
No	22 (32.8)	8 (42.1)	14 (29.2)	
Unknown	36 (53.7)	10 (52.6)	26 (54.2)	
Taste disorder, yes, n (%)	8 (11.9)	6 (31.6)	2 (4.2)	0.005
Stomatitis, yes, n (%)	4 (6.0)	1 (5.3)	3 (6.3)	1.000
Nausea, yes, n (%)	1 (1.5)	1 (5.3)	0 (0.0)	0.284
Vomiting, yes, n (%)	1 (1.5)	1 (5.3)	0 (0.0)	0.284
Edema, yes, n (%)	2 (3.0)	1 (5.3)	1 (2.1)	0.490
Oral nutritional supplement, yes, n (%)	14 (20.9)	9 (47.4)	5 (10.4)	0.002
Nutritional infusion, yes, n (%)	1 (1.5)	0 (0.0)	1 (2.1)	1.000
Topical medication for stomatitis, yes, n (%)	4 (6.0)	1 (5.3)	3 (6.3)	1.000
Mouthwash prescribed, yes, n (%)	3 (4.5)	0 (0.0)	3 (6.3)	0.553
Oral mucosal-protective medication, yes, n (%)	17 (25.4)	5 (26.3)-	12 (25.8)-	1.000-
Analgesics for stomatitis, yes, n (%)	3 (4.5)	0 (0.0)	3 (6.3)	0.553
Anamorelin, yes, n (%)	10 (14.9)	5 (26.3)	5 (10.4)	0.132
Regular oral corticosteroid therapy, yes, n (%)	9 (13.4)	1 (5.3)	8 (16.7)	0.427
NSAIDs, yes, n (%)	22 (32.8)	5 (26.3)	17 (35.4)	0.475
Aspirin (antiplatelet), yes, n (%)	6 (9.0)	1 (5.3)	5 (10.4)	0.667
Gastric medication, yes, n (%)	41 (61.2)	13 (68.4)	28 (58.3)	0.445
Oral zinc supplement, yes, n (%)	1 (1.5)	0 (0.0)	1 (2.1)	1.000

*BWL* Body weight loss, *PS* Performance status, *Sm* Small cell carcinoma, *Ad* Adenocarcinoma, *Sq* Squamous cell carcinoma, *IQR* Interquartile range, *N* Number, *ICI* Immune checkpoint inhibitors, *BMI* Body mass index, *CRP* C-reactive protein, *CA* Cytotoxic anticancer drugs, *EGFR* Epidermal growth factor receptor, *IQR* Interquartile range, *CRP* C-reactive protein, *PS* Performance status, BMI Body mass index, *OR* Odds ratio, *CI* Confidence interval, *CR* Complete remission, *PR* Partial remission, *SD* Stable disease, *PD* Progressive disease

*Dental visit within the past six months before oral frailty assessment

**All chemotherapy regimens (CA་ICI) in this study were platinum-based

***All CA regimens were composed of non-platinum cytotoxic agents

****In the CA + molecular targeted therapy group, all regimens included non-platinum cytotoxic agents

Adenocarcinoma was the most common histology (n = 29, 43.3%). Stage IV was the most common cancer stage (n = 33, 49.3%). First-line chemotherapy was administered to 23 patients (34.4%), and adjuvant chemotherapy to 14 (20.9%). Regimens including immune checkpoint inhibitors were used in 34 (50.7%), and platinum-based combination therapy in 12 (17.9%). At the time of oral frailty assessment, disease status based on RECIST v1.1 was partial response (PR) in 40 patients (59.7%), stable disease (SD) in 14 (20.9%), and progressive disease (PD) in 13 (19.4%).

### Univariable analyses (Table 2)

Weight loss ≥5% within the prior 6 months was observed in 19 patients (28.4%). Patients were classified into the body weight loss (BWL) group (n = 19) and the non-BWL group (n = 48). Age, sex, stage, disease status, and treatment type did not differ significantly between groups. ECOG PS was significantly poorer in the BWL group (p < 0.001), and BMI was significantly higher (p = 0.020). The BWL group had a higher prevalence of low grip strength (73.7% vs 27.1%, p < 0.001), appetite loss (57.9% vs 20.8%, p = 0.003), elevated CRP (median [IQR]: 1.12 [0.38–3.41] vs 0.23 [0.09–0.79], p = 0.004), positive OF-5 (84.2% vs 31.3%, p < 0.001), and dysgeusia (31.6% vs 4.2%, p = 0.005), and more frequently received prescriptions for oral nutritional supplements (47.4% vs 10.4%, p = 0.002). There were no significant between-group differences in dental visits; presence of oral mucositis or periodontitis; denture use or ill-fitting dentures; nausea; vomiting; edema; or the use of topical/oral mucosal agents, analgesics for mucositis, NSAIDs, steroids, aspirin, zinc preparations, or anamorelin.

### Multivariable analyses

Multivariable logistic regression models were constructed with oral frailty and dysgeusia as the primary exposures, with stepwise adjustment for PS, CRP, sex, and cancer stage (Table3).

**Table 3:** Results of multivariable analysis

	ModelⅠ			Model Ⅱ		Model Ⅲ	
Parameters (reference)	OR (95% CI)	p-value	OR (95% CI)		p-value	OR (95% CI)	p-value
Oral frailty, yes (no)	28.79 (1.81–36.05)	0.006	19.32 (1.33 – 194.82)		0.006	21.31(1.49–374.58)	0.002
Taste disorder, yes(no)	39.84 (1.76–40.62)	0.001	24.88 (0.82–149.79)		0.011	23.04 (0.57–295.71)	0.003
PS, ≥ 2 (< 2)			4.46 (0.55–34.48)		0.001	5.51 (0.59–42.69)	0.002
CRP			1.02 (−0.32–32.45)		0.917	0.96 (−0.59–56.56)	0.872
Sex						1.05 (−18.76–20.89)	0.753
Advanced Stage, yes (no)						0.37 (−42.05–15.55)	0.328

*CRP* C-reactive protein, *PS* Performance status, *BMI* Body mass index,* OR* Odds ratio, *CI* Confidence interval

Advanced Stage was defined as Stage III, Ⅳ, and recurrence

#### Model 1 (oral frailty + dysgeusia)

Oral frailty (OR:28.79:95% CI: 1.81–36.05, p = 0.006) and dysgeusia (OR:39.84,95% CI: 1.76–40.62, p = 0.001) were both significantly associated with weight loss.

#### Model 2 (+ PS and CRP)

After adding PS and CRP, oral frailty (OR:19.32CI: 1.33–194.82, p = 0.006) and dysgeusia (OR:24.88CI: 0.82–149.79, p = 0.011) remained significant.

#### Model 2 (+ PS and CRP)

After adding PS and CRP, oral frailty (OR:19.32CI: 1.33–194.82, p = 0.006) and dysgeusia (OR:24.88CI: 0.82–149.79, p = 0.011) remained significant.

### Model 3 (+ sex and stage)

After further adjustment for sex and stage, oral frailty (OR:21.31 95% CI: 1.49–374.58, p = 0.002) and dysgeusia (OR:23.04 95% CI: 0.57–295.71, p = 0.003) remained significant.

Variance inflation factors were <3 in all models, indicating minimal multicollinearity.

## Discussion

This study examined factors associated with ≥5% weight loss over 6 months among patients with lung cancer undergoing outpatient chemotherapy. In multivariable analyses with stepwise adjustment for PS, CRP, sex, and stage both oral frailty (assessed using the OF-5) and dysgeusia were associated with weight loss. Although both conditions are common in patients undergoing chemotherapy, they may be underrecognized or underdiagnosed in routine practice [19.29–30]. Our findings suggest that declines in oral function and taste abnormalities may contribute to reduced intake and malnutrition, thereby promoting weight loss. Early identification may therefore be clinically valuable.

Weight loss in cancer reflects not only tumor-related metabolic alterations and inflammatory responses, but also treatment-related toxicities, such as mucositis, taste and olfactory changes, and nausea. These factors may collectively disrupt energy balance and adversely affect QOL and survival [31]. In patients with lung cancer, baseline weight loss at treatment initiation has been associated with increased chemotherapy toxicity and shorter survival [32–34]. Thus, early detection of nutritional decline during treatment may be crucial for improving outcomes and maintaining treatment continuity.

Oral frailty refers to mild, reversible decline in oral function that can lead to monotonous diets and reduced intake due to fewer remaining teeth, difficulty with mastication or swallowing, xerostomia, and impaired articulation. This process may progress from reduced intake to weight loss and subsequent decline in physical function and systemic frailty. Oral functional decline may thereby act as both a cause and consequence of systemic frailty [13]. The higher prevalence of OF-5 positivity in the weight-loss group aligns with this theoretical framework and supports a pathway in which oral function influences eating behavior and nutritional status. As this was an observational study, causal inferences cannot be made, and interpretations should be limited to associations.

The OF-5—proposed in a 2024 joint statement by three Japanese societies [7, 25]—is a simple screening tool that can be administered by non-dental clinicians without specialized equipment or dental oversight. It is therefore feasible for use in oncology clinics. Our findings provide real-world support for the practicality of OF-5-based screening in non-dental settings. Future research should incorporate objective assessments made by dental professionals (e.g., occlusion, tongue pressure, masticatory performance) to more comprehensively examine oral function and nutritional status.

Dysgeusia is also a common adverse event in oncology practice and has been reported to reduce intake and alter food preferences, contributing to weight loss [29, 35]. A scoping review in lung cancer populations reported dysgeusia in 35–38% of patients before treatment and 35–69% during treatment, with consistent associations with reduced intake, weight loss, and poor QOL [36]. Dysgeusia is common across regimens, particularly those including platinum, and has been closely linked to weight loss and QOL decline [37]. Proposed mechanisms include suppression of basal cell proliferation and delayed taste bud regeneration, peripheral neuropathy, hyposalivation, mucositis, and elevated levels of inflammatory cytokines [30]. Interventions such as zinc supplementation (intravenous or oral) and polaprezinc (zinc-L-carnosine) have been reported to improve taste scores and mucositis in some studies, although preventive effects remain uncertain and consensus is lacking [38, 39]. The recent Oncology Nursing Society guideline recommends combining selective pharmacologic approaches with non-pharmacologic, multidisciplinary strategies such as texture/temperature adjustments, taste and olfactory training, xerostomia management, and nutrition counseling [30]. The observed association between dysgeusia and weight loss in our cohort underscores the importance of such comprehensive supportive care.

The finding that BMI was higher in the weight-loss group requires careful interpretation. Although an “obesity paradox”—wherein higher BMI is associated with better outcomes—has been reported in patients with cancer [40], these observations may reflect the relative balance of muscle and fat mass rather than adiposity alone. Sarcopenic obesity, characterized by low skeletal muscle mass despite elevated BMI, has been linked to poorer outcomes [41]. Moreover, BMI may function as a mediator in the pathway from decreased intake to weight loss; adjusting for BMI could therefore underestimate total effects.

Accordingly, we considered BMI as a biological background factor and excluded it from the primary models. Future studies incorporating CT-based muscle measurements and inflammatory markers could provide more accurate assessments of body-composition changes.

### Limitations

This study has some limitations. First, it was a single-center retrospective study with a limited sample size, resulting in a low events-per-variable ratio (EPV = 2.4) and a potential risk of overfitting and estimation bias. To mitigate these concerns, we restricted the primary models to clinically important variables, applied stepwise adjustment, and evaluated robustness using 1,000-iteration bootstrap resampling. However, although bootstrap resampling can help quantify uncertainty, it does not eliminate estimation bias; therefore, casual inferences cannot be made. Some models showed wide confidence intervals, particularly those involving rare events, suggesting possible quasi-separation and inflated standard errors. Because the Firth penalized likelihood correction could not be implemented due to system constraints, this limitation should be considered when interpreting the results. In some models, the p-value was below 0.05 while the 95% bootstrap confidence interval marginally included 1, reflecting methodological differences between the Wald-based p-values and percentile or BCa bootstrap intervals. The Results section notes that these findings should be interpreted with caution. Prospective validation with larger samples and the use of penalized estimation methods (e.g., Firth regression) would strengthen the evidence. Second, oral mucosal conditions (e.g., mucositis) are important confounders of weight loss, but standardized visual assessments and pain scores were not consistently available due to the retrospective design. We extracted proxy indicators (dysgeusia, prescription of topical agents, or analgesics) and adjusted for dysgeusia as a key covariate; however, residual confounding from unmeasured mucosal severity cannot be excluded. If patients with severe mucosal injury were more likely to have higher OF-5 scores and greater weight loss, the association between OF-5 and weight loss may have been overestimated. Third, a concurrent dental examination was not performed at the time of oral frailty assessment, and comprehensive dental evaluation data (e.g., mucosal status, occlusion, tongue pressure) were not available for all patients. Because many patients received dental care outside our institution, dental-visit history was not used as a principal confounder due to incomplete ascertainment. Instead, we analyzed chart-recorded indicators related to oral symptoms and treatment. Although the OF-5 was administered by non-dental clinicians, consistent with its intended use as a screening tool, incorporating dental professional assessments would enhance measurement validity. Future studies should involve multidisciplinary collaboration and objective oral function assessments. Fourth, cancer cachexia is a multifactorial syndrome involving tumor progression, metabolic abnormalities, inflammation, and reduced intake. Because its diagnostic criterion (≥5% weight loss over 6 months) overlaps with our outcome definition [26], analyzing cachexia as an explanatory variable would introduce definitional circularity. Future prospective studies incorporating muscle mass and metabolic indices are needed to clarify the interrelationships among weight loss, cachexia, oral frailty, and dysgeusia. Fifth, appetite was assessed using a single item corresponding to item A of the Mini Nutritional Assessment–Short Form (MNA-SF), rather than the full MNA-SF. This item may reflect influences beyond appetite (e.g., gastrointestinal symptoms or dysphagia) and should therefore be interpreted as a supplementary indicator. Future research should consider validated appetite-focused scales, such as the Simplified Nutritional Appetite Questionnaire. Finally, although BMI is an important indicator of baseline nutritional status, it may lie on the causal pathway from decreased intake to weight loss. Adjusting for BMI may therefore lead to overcontrol. Given EPV constraints, BMI was excluded from the primary models and treated as a biological background factor. Larger studies are needed to reassess models that incorporate BMI as an adjustment variable.

## Conclusions

Among patients with lung cancer receiving outpatient chemotherapy, oral frailty (assessed using the OF-5) and dysgeusia were associated with weight loss. Early screening for oral frailty using the OF-5, which can be implemented by non-dental clinicians, may help identify patients at risk of nutritional decline. Multidisciplinary supportive care that includes assessment and management of oral function and taste disturbances appears warranted in oncology practice. Collaboration with dental professionals and incorporation of standardized oral and taste assessments in future studies will be important for confirming the reproducibility of these findings and evaluating the potential benefits of targeted interventions.

## Data Availability

The data that support the findings of this study are available from the first author (U.K.) upon reasonable request.

## Abbreviations

OF5: Oral Frailty 5-item checklist
MNA-SF: Mini Nutritional Assessment-Short Form
OR: odds ratio
CI: confidence interval
BMI: body mass index
CRP: C-reactive protein
PS: Performance Status
BWL: body weight loss
Sm: small cell carcinoma
Ad: adenocarcinoma
Sq: squamous cell carcinoma
IQR: interquartile range
N: number
ICI: immune checkpoint inhibitors
CA: cytotoxic anticancer drugs
EGFR: epidermal growth factor receptor
IQR: interquartile range
CR: complete remission
PR: partial remission
SD: stable disease
PD: progressive disease
